# Use of external evidence for design and Bayesian analysis of clinical trials: a qualitative study of trialists’ views

**DOI:** 10.1186/s13063-021-05759-8

**Published:** 2021-11-08

**Authors:** Gemma L. Clayton, Daisy Elliott, Julian P. T. Higgins, Hayley E. Jones

**Affiliations:** 1 Department of Population Health Sciences, Bristol Medical School, University of Bristol, Bristol, UK; 2 Bristol Centre for Surgical Research, Population Health Sciences, Bristol Medical School, University of Bristol, Bristol, UK; 3 NIHR Applied Research Collaboration West (ARC West) at University Hospitals Bristol and Weston NHS Foundation Trust, Bristol, UK

**Keywords:** Evidence synthesis, Bayesian analysis, Trials, Qualitative, Informative prior distributions, Meta-epidemiology

## Abstract

**Background:**

Evidence from previous studies is often used relatively informally in the design of clinical trials: for example, a systematic review to indicate whether a gap in the current evidence base justifies a new trial. External evidence can be used more formally in both trial design and analysis, by explicitly incorporating a synthesis of it in a Bayesian framework. However, it is unclear how common this is in practice or the extent to which it is considered controversial. In this qualitative study, we explored attitudes towards, and experiences of, trialists in incorporating synthesised external evidence through the Bayesian design or analysis of a trial.

**Methods:**

Semi-structured interviews were conducted with 16 trialists: 13 statisticians and three clinicians. Participants were recruited across several universities and trials units in the United Kingdom using snowball and purposeful sampling. Data were analysed using thematic analysis and techniques of constant comparison.

**Results:**

Trialists used existing evidence in many ways in trial design, for example, to justify a gap in the evidence base and inform parameters in sample size calculations. However, no one in our sample reported using such evidence in a Bayesian framework. Participants tended to equate Bayesian analysis with the incorporation of prior information on the intervention effect and were less aware of the potential to incorporate data on other parameters. When introduced to the concepts, many trialists felt they could be making more use of existing data to inform the design and analysis of a trial in particular scenarios. For example, some felt existing data could be used more formally to inform background adverse event rates, rather than relying on clinical opinion as to whether there are potential safety concerns. However, several barriers to implementing these methods in practice were identified, including concerns about the relevance of external data, acceptability of Bayesian methods, lack of confidence in Bayesian methods and software, and practical issues, such as difficulties accessing relevant data.

**Conclusions:**

Despite trialists recognising that more formal use of external evidence could be advantageous over current approaches in some areas and useful as sensitivity analyses, there are still barriers to such use in practice.

**Supplementary Information:**

The online version contains supplementary material available at 10.1186/s13063-021-05759-8.

## Background

The importance of using existing evidence to inform the design and analysis of a randomised controlled trial (RCT) is increasingly recognised by trialists and funders alike [1]. Intuitively, new research should learn from previous, related research and avoid unnecessary repetition and research waste [2, 3]. Evidence syntheses now play an important role [4]. Results from systematic reviews are often used informally in planning new trials, for example, to indicate whether a gap in the current evidence base justifies a new trial [5, 6].

A more formal approach to using existing evidence is to use a Bayesian statistical framework in which previous evidence about a parameter is used to derive a prior distribution [7]. Parameters that might be informed by a synthesis of previous evidence include: the intervention effect, either at the design stage only (in power or sample size calculations [8–11]) or at the analysis stage [12]; the control group event rate, particularly for rare outcomes [13, 14]; and the intra-class correlation coefficient in a cluster RCT [15]. Furthermore, incorporating external evidence from ‘meta-epidemiological’ studies about the extent of bias typically associated with potential methodological limitations in the new trial, e.g. infeasibility of blinding the patient or personnel [16, 17], can produce a bias-adjusted treatment effect estimate [18], allowing the analyst to assess the robustness of their findings [19]. Although studies suggest evidence synthesis is increasingly used to inform trial design, the extent to which Bayesian approaches are used, and opinions on their use, is unclear [19, 20]. In a recent survey of attendees of the International Clinical Trials Methodology Conference, many responders indicated they felt increased use of external evidence in trial design and analysis would be desirable [20].

We undertook a qualitative study to explore trialists’ views on, and any experiences with, incorporation of external evidence through a Bayesian statistical approach. We focused on the use of a synthesis of relevant evidence on particular parameters, with reference to examples which we described, and identification of any barriers to the use of these approaches. We explored which types of external evidence were considered most potentially relevant and useful, and the likely acceptability of such use in practice.

## Methods

Ethical approval was obtained by the University of Bristol on 27 April 2017 (Reference number 48101). We report our study according to the Consolidated Criteria for Reporting Qualitative Research (COREQ) [21] (summary table in Table S1).

### Recruitment and sampling

We aimed to sample a range of individuals, from trials units and universities across multiple locations in England, with experience of working in trials. The following positions were targeted to ensure a diverse sample: methods leads, lead trial statisticians, trialists writing grant applications and leads of NIHR funded trials. Both clinicians and statisticians were included to capture a range of perspectives. Individuals involved in developing evidence synthesis methods were excluded, as we felt these methodologists may tend to be more supportive of using advanced methods than most trialists [20]. We did not require participants to have experience with or any knowledge of Bayesian analysis.

A key informant sampling approach was initially adopted [22], whereby an initial list of potential participants was drawn up from individuals known to the study team (GLC, HEJ, JPTH). The lead researcher (GLC) contacted these via email, to explain the study purpose through the provision of a participant information sheet (PIS) and to ask whether they would be willing to take part in an interview. We did not specifically refer to Bayesian analysis in the PIS. Instead, the PIS explained that we wanted to explore ‘trialists’ views and experiences of analysing trials in the context of the wider evidence base’.

After the initial list of potential participants had been approached, participants were then identified via snowball sampling [22, 23], whereby interviewees suggested potential contacts, supplemented by purposive sampling [24, 25] to ensure we sampled from our entire intended population, for example, including both junior roles, such as trial statisticians, and more senior roles [26]. Recruitment was driven by theoretical saturation (whereby data collection continues until no new themes emerged) [27] and when it was felt that maximum variation had been reached [28, 29].

### Data collection

Interviews were semi-structured to ensure similar areas were covered in each interview, with sufficient flexibility to allow new issues of importance to emerge [30]. There were separate topic guides for clinicians and statisticians. These were very similar, with some questions rephrased for clinicians to focus more on the conceptual ideas of using previous evidence in different scenarios. The topic guides were initially developed with suggestions from all members of the study team and were iteratively modified in light of emerging findings. An example of a topic guide used is shown in Figure S1. We began the interviews by trying to elicit which statistical methods trialists had used. We then explored how previous evidence was considered and/or used, when designing and analysing a trial. We further explored participants’ views on, and any experiences with, the formal incorporation of existing data via Bayesian approaches. The basic concepts of a Bayesian approach were first explained to participants who were not familiar with this.

The latter part of each interview was used to examine three hypothetical scenarios in which evidence syntheses could be incorporated into a trial. We focussed on the use to inform the following parameters: (1) the intervention effect,  (2) potential bias associated with unavoidable trial limitations and (3) ‘nuisance’ parameters such as baseline rates of adverse events. We also explored the potential barriers to implementing these methods in practice. Interviews were recorded using an encrypted audio recorder.

### Data analysis

Interview recordings were transcribed verbatim whole to conduct a comprehensive analysis. Transcripts were analysed thematically and inductively by GLC (the lead investigator with a background in statistics and experience working as a trial statistician), under the guidance of DE (an experienced qualitative researcher), using techniques of constant comparison whereby similarities and differences between interviewees were explored [31]. Coding was conducted using the qualitative data analysis software, NVivo (Version 11). Codes within transcripts were analytically summarised such that each code could be interpreted on its own [31], collated to explore any emerging patterns and organised into themes. Emerging themes were compared with other codes across the dataset, to see if there were any shared or disparate views amongst particular subgroups [28], such as methods leads, lead statisticians or trialists within the same unit [32].

The first three transcripts were double coded by DE, and a further three transcripts were double coded by a member of the study team with expertise in statistics and evidence synthesis (HEJ). The overall meaning and interpretation of codes were found to be similar, and minor disparities were discussed until there was a consensus. After analysing the first 13 interviews, we felt that maximum variation had been reached [28]. We conducted a further three interviews to check that no new codes emerged directly relating to the key findings.

## Results

### Participants

Of those individuals approached, only one declined. Amongst the 16 interviewees, three had a clinical background (two of whom were Chief Investigators), while 13 had a statistics background. 25% (4/16) had greater than 10 years of experience working in trials. Interviews lasted a mean of 54 minutes (range 37–79). Table 1 provides participant and employment role-related characteristics.

**Table 1:** Participant and employment role-related characteristics

ID	Job role	Role description	Years in career	Types of trials	Highest educational qualification
*Senior stat*, P1	Trial statistician	Conducting analysis of trials in practice	3.5	RCTs, feasibility studies, primary and secondary care	MSc Statistics
*Senior stat*, P2	Senior statistician	Lead trial statistician and writing grant applications	6	RCTs, observational studies, surgery	MSc Statistics
Ch inv, P3	Medical professor	Responsible for people planning/conducting the analysis of a clinical trial and lead of NIHR funded trials	> 10	RCTs, FIH, translational studies	PhD, Clinical
*Methods lead*, P4	Principle statistician	Responsible for people planning/conducting the analysis of a clinical trial and lead of NIHR funded trials	> 10	RCTs, feasibility studies	PhD, Statistics
Ch inv, P5	Medical professor	Responsible for people planning/conducting the analysis of a clinical trial and lead of NIHR funded trials	> 10	RCTs, FIH, translational studies	MD, Clinical
Clin stud, P6	Clinical PhD student	Conducting analysis of trials in practice	5	RCTs	PhD, Clinical
*Trial stat*, P7	Trial statistician	Conducting analysis of trials in practice	5	RCTs, feasibility studies	MSc Statistics
*Trial stat*, P8	Trial statistician	Planning and conducting the analysis of a clinical trial	3.5	RCTs, feasibility studies	PhD, Statistics
*Trial stat*, P9	Trial statistician	Conducting analysis of trials in practice	3.5	RCTs, feasibility studies, primary and secondary care	MSc Statistics
*Methods lead*, P10	Principle statistician	Responsible for people planning/conducting the analysis of a clinical trial	> 10	RCTs, feasibility studies	MSc Statistics
*Senior stat*, P11	Senior statistician	Planning and conducting the analysis of a clinical trial	7	RCTs, feasibility studies	MSc Statistics
*Senior stat*, P12	Senior statistician	Conducting analysis of trials in practice	4	RCTs, feasibility studies	MSc Statistics
*Senior stat*, P13	NIHR Research Fellow	Conducting analysis of trials in practice	0.5	RCTs, feasibility studies, surgery	MSc Statistics
*Senior stat*, P14	Senior statistician	Planning and conducting the analysis of a clinical trial and writing grant applications	6.5	RCTs, feasibility studies, observational data/cohorts	MSc Statistics
*Methods lead*, P15	Principle statistician	Planning and conducting the analysis of a clinical trial and writing grant applications	5	RCTs, observational data/cohorts	MSc Statistics
*Senior stat*, P16	Senior statistician, PhD student	Conducting analysis of trials in practice	5	RCTs, feasibility studies	MSc Statistics

*RCTs* randomised controlled trials; MSc Master of Science; NIHR National Institute for Health Research; FIH First in human; PhD Doctor of Philosophy; MD Doctor of Medicine

### Analysis

We report our findings in four themes, supported by quotations.

#### Theme 1: External evidence is used informally in trial design but not in analysis

Participants across all trials units were using evidence syntheses informally in a number of ways to inform trial design. Uses of evidence were included to justify the trial and inform the choice of outcomes or parameter values for sample size calculations.

Senior stat, P12: "*I suppose it’s something that people do informally but not in a structured Bayesian [way].*"

Senior stat, P1: "*When I was determining a minimal clinically important difference I had, you have various methods to determine a minimal and clinically important difference. I basically did a meta-analysis of all of those results and came up with a value and that’s the value I reported.*"

Participants reported that the sourcing of previous evidence was often instigated by the clinician, who would share a published paper, usually a systematic review, with the study team. This was typically used to support the need for the trial and to demonstrate equipoise in funding applications.

Ch inv, P5: "*Obviously, there’s the evidence of equipoise. There’s the evidence of the knowledge gap.*"

Ch inv, P3: "*And unless you can convince a funder or ethics committee of equipoise to two treatment arms then you won’t get, you can’t do the study. There’s got to be some sort of uncertainty.*"

All sixteen trialists reported that they consistently used ‘standard’ and ‘simpler’ statistical models such as logistic, linear and Cox regression, or mixed effect models for repeated measure data, that did not incorporate external evidence through a Bayesian analysis.

Methods lead, P10: "*It’s generally in a way just the simplest technique that will get the job done and not overcomplicating it. Calculations get confused enough as it is!"*

Some participants indicated that they had used previous studies informally to inform the choice of statistical methods for their analysis.

Methods lead, P4: "*I don’t think I’ve ever used previous evidence in the analysis stage other than if I was looking it up before I did the analysis to inform what analysis I might do, but not actually [using previous evidence]."*

Participants indicated that any external information on adverse event rates is summarised descriptively.

Methods lead, P10: "*I rely on the DMC [Data monitoring commitee] quite a lot basically. I don’t think we’ve got good methods for looking at adverse event rates really. It’s often just listings or tabulations."*

Senior stat, P1: "*I think a lot of the adverse events that I’ve reported, we then compare them to cohort data or kind of a population level data rather than trial specific."*

None of our participants had used external evidence to adjust for potential biases in their numerical results. Trialists indicated they would simply describe limitations in their trial design, e.g. inability to blind outcome assessors, in their discussion.

Senior stat, P1: "*Yes, I suppose it can do [bias the results] but I’ve never been particularly worried about it.…I haven’t done it [bias adjustment]; not thought about it or even something we discuss as being potentially biased; just see it as a limitation."*

#### Theme 2: Concerns regarding the acceptance of Bayesian methods in practice

Some senior statisticians, particularly methods leads, voiced concerns that trials using Bayesian methods might be less likely to be published or funded.

Methods lead, P4: "*Whether it would be accepted by decision makers. I guess if you’re doing anything that’s not the norm, you’d just be a bit scared, even getting it published. Reviewers could be like ‘what on earth have you done? I’ve worked in trials all my life and I’ve never done this."*

Some also felt that Chief Investigators, who ultimately sign off the analysis plan, would not understand Bayesian methods and, moreover, would not encourage their use.

Senior stat, P11: "*I think actually clinicians and things are more familiar with the frequentist approach rather than Bayesian, and actually it can be more difficult when you say, ‘I’ve used Bayesian methods,’ and they think, ‘Oh, what have you done?’"*

There was also a reluctance to change current methods.

Senior stat, P1: "B*ut I think you’d have to do a lot, change a lot of peoples mind set to be able to make them do this type of analysis compared with the analysis we already do."*

All 16 participants also indicated that they felt it was difficult to trust external evidence and expressed concerns that incorporating it in their analysis could bias their own trial results. For example, participants were concerned about likely differences in the population between the external evidence and their own trial.

Senior stat, P11: "*I am always a bit uncertain with meta-analysis about how you can group together different trials, because they are different trials. They don’t use the same patient groups and there are different intricacies in there. I suppose it depends on the call to the evidence and if there were reasons why that cohort were different, or the outcomes were different, or the intervention was different."*

#### Theme 3: Practical challenges of use

Throughout the interviews it was clear there were practical challenges participants felt they (and/or the wider trials unit) would face if they wanted to use external evidence formally in practice. One of the most common issues surrounded logistics and the administrative burden associated with accessing external data, and the corresponding consideration of anonymisation of research participants.

Trial stat, P8: "*I suppose if there was, if there was consistency in the way the studies were reported and there was a way, a simple way of collecting all of the high-quality evidence together very quickly, then that would obviously be a big help but yes, I suppose that’s a bit of a pipe dream really."*

Methods lead, P10: "*If it’s publicly funded you need to make the data available and that seems reasonable. But there’s still always an administrative exercise in getting through approvals and getting that, and for somebody to create a dataset that can be shared without risking identifiable data."*

Most participants thought a systematic review was the most obvious source of external data. However, most indicated that their trial units did not have direct access to a systematic review team, and they viewed systematic reviewers as having a different skill set from their own.

Ch Inv, P5: "*One of the problems is there’s probably a shortage of systematic review capacity. So, finding systematic reviewers is really tough actually."*

More senior trial members identified concerns regarding how much extra time would be needed to implement Bayesian methods and the implicit costs associated with this.

Ch Inv, P3: "*I’m trying to get funding for a study now to do this comparison, I can’t easily spend loads of money having a statistician spending ages trying to make a brilliantly efficient trial design..."*

It was frequently brought up that, in order for these methods to be used in practice, there would need to be guidelines and requirements by funders.

Trial stat, P8: "*So, I think that would be a helpful if there was, I mean certainly if there was some sort of guidance that had been produced elsewhere."*

Methods lead, P10: "*I think that’s partly about real world examples that you can look at and see how somebody did it and then it’s also about software and knowing how to implement it even if you wanted to."*

Many statisticians also did not feel confident in using Bayesian methods or Bayesian statistical software. They expressed concerns about the time and financial pressures associated with having to learn these techniques.

Senior stat, P11: "*I personally don’t go anywhere near them [Bayesian methods]. I think I did do a course in Bayesian stuff, but I just don’t think I work that way and I don’t feel comfortable using Bayesian methodology, so I personally would shy away from it."*

Trial stat, P9: "*It [Bayesian analysis] was a black-box moment of it went into the system and came out and I didn’t really know what had gone on in between [laughs]. Very bad statistician!"*

Methods lead, P4: "*I think a lot of it is accessibility of the software because Stata it’s just very straightforward, WinBUGS it’s not. So, I think that’s a massive hurdle. If you could do it in Stata people would probably do it."*

#### Theme 4: Perceived advantages of making use of existing evidence

Despite the barriers described in themes 2 and 3, many of the trialists expressed enthusiasm for the concept of making more use of existing data in particular as secondary or sensitivity analyses.

Senior stat, P16: "*OK, as a statistician, you’re always taught the more data you have, the better. The more information you have the better. So why – if you’ve got the information there – would you not use it?"*

Senior stat, P1: "*… I think it would take a lot, as a said before, a lot for people to change the specific analysis they were going to do. So I think this would be a subsequent or secondary analysis that people would do but it would be interesting."*

Many thought that making more use of existing data would be advantageous, as a lot of time and money is invested in trials, which it is important not to waste.

Senior stat, P1: "*We don’t want to do a trial that wastes a) time and b) money, so if we had existing evidence which would cut down time and money then I think we should do it to start with."*

Senior stat, P11: "*I guess, because you do collect a heck of a lot of data for each trial and obviously not everyone has registry databases like ourselves, I don’t know if there is some way that there will always be like an evolving cycle of data. …. I think I’ve heard it talked about the trial in a certain area you always collect certain variables and then those variables could be uploaded to a dataset and then it actually creates a big one. Everyone’s trial data gets compiled together and then you do have a big database that you could then use to inform sample size calculations and other things like that."*

We observed that when trialists think of Bayesian methods, they were generally only thinking about prior information on the treatment effect—and may be unaware of available methods to make use of data on other parameters. In discussing the potential to use external evidence to inform other types of parameters (see the ‘Methods’ section), several trialists expressed interest in the idea of making better use of existing safety data so that rare events could be picked up faster. When asked about their views on using data from a similar population to predict what the expected adverse event rate would be in the control arm, many felt this could be potentially advantageous. This was also similar to what some trialists were doing informally.

Ch Inv, P3: "*So, we do make use of it [external evidence on adverse event rates] but obviously in a suboptimal way and I can imagine that doing this kind of approach for adverse events for example. would offer greater safety would allow safety signal to become obvious in my study earlier maybe so therefore better."*

The concept of using an existing meta-analysis to power a new trial, based on its ability to impact an existing meta-analysis [8, 10, 11, 33], was unfamiliar to all participants. Having briefly explained the concepts of this to participants, many thought it was an attractive idea and could make the trial more efficient.

Methods lead, P4: "*If you’re wanting to change practice, your one trial is not going to change practice. The body of evidence [meta-analysis] is going to change practice."*

Senior stat, P1: "*You’re not gonna sort of waste time and money showing an effect size in a single trial when you might be able to do it in a combination with existing studies. I think that’s quite sensible, but I guess it’s a case-by-case basis."*

Trial stat, P8: "*I think it’s quite sensible probably to power it based on, you know, making a change to that if it means that you, you know, you’re gonna recruit less participants."*

## Discussion

We found that trialists were using existing evidence in many ways, including to justify there is a gap in the evidence base and to inform parameters in sample size calculations. However, none of the trialists in our sample had an experience of explicitly incorporating prior information in either trial design or analysis through the use of Bayesian statistical methods. Our study showed that trialists felt they could be making more use of existing data to inform the design and analysis of a clinical trial in particular scenarios, such as in secondary or sensitivity analyses. We observed that when trialists thought of Bayesian methods, they appeared to be thinking about the use of prior information on the treatment effect only and may be unaware of available methods to make use of data on other parameters. Several participants in our study found the idea of formal use of evidence on other parameters appealing and thought improvements could be made to current methods. The use of informative priors based on external evidence in sample size calculations and to assess whether adverse event rates were higher than baseline was perceived as attractive. Similarly, although trialists do not think about how their trial results will influence a future meta-analysis, many thought it would be useful to investigate when conducting the sample size calculation. In contrast, no participant expressed enthusiasm for the concept of ‘bias adjustment’ based on external evidence. Participants also identified barriers to implementing these methods in practice. Trialists expressed concerns about the relevance or quality of external data and how incorporation of this could potentially affect their own trial. We also found that trialists did not feel confident in the use or interpretation of Bayesian methods and identified practical issues including difficulty accessing relevant data, anonymisation issues and the extra time associated with this.

Our finding regarding the relevance of prior information being a key concern to trialists is consistent with the results from a recent survey of trial methodologists [20] and has been an area greatly discussed [34]. The greatest barrier to the use of existing evidence according to this survey was time constraints. Although we did not identify time constraints as an overarching theme, this issue was mentioned by some participants as one of the practical challenges of using external evidence in trials. A more detailed exploration in our study revealed the extra time needed to conduct a systematic review was a concern, given that systematic review teams are often not integrated into clinical trial teams. We also found trialists found it difficult to access and collate other external data, either aggregate or individual participant data. Some statisticians noted the time that would be needed to learn new methods and software. Lilford [35] argues the assumption of equipoise in randomised trials is misleading to the patients being invited to participate in a new trial. More often than not, some evidence exists before a trial such as similar treatments in the same disease area or the same treatments in other populations. The findings of our qualitative study are consistent with this: trialists recognised that previous studies gave some indication about the potential intervention effect and that it is the accumulation of evidence that is likely to change practice. However, they did not explicitly incorporate this information into the trial design or analysis. Our study appears consistent with Brocklehurst et al. [36] in the overarching finding that it remains unclear to trialists (including investigators) the process by which external evidence should be considered, and at precisely which stages of a trial.

Having found that the statisticians in our sample did not feel confident in the use of, and/or had concerns about the acceptability of, Bayesian methods, more training and specific methodological guidelines on the use of Bayesian approaches in trials may be beneficial. In particular, guidance could raise awareness of Bayesian approaches to incorporate external evidence on parameters other than the treatment effect, given that our participants knew little about options in this area. Tutorial papers, user-written packages in generic rather than specialist software and provision of example code may increase accessibility. We note that Bayesian approaches are becoming easier to implement in practice via, for example, the development of core R packages [37]. Development of an easily accessible repository of relevant data, ideally individual participant data, would help facilitate the use of external evidence in practice. There have been many calls for such a platform [38].

One limitation of this study is that snowball sampling was used to identify participants after initial key contacts in each group were sampled from colleagues known to the study team. This potentially limits the generalisability of the findings to all trialists. However, as data collection continued, sampling became increasingly purposive, with a view to achieving a sample of maximum variation, to ensure insights were captured from a range of informants operating in different contexts [29]. We succeeded in sampling from each group of our intended population [29, 39, 40], including both statisticians and clinicians in a range of senior and junior positions. We also acknowledge that our sample size of 16 participants may be limited; however, qualitative studies are typically small in order to generate rich and in-depth insights and accounts of participants’ views and experiences [40, 41]. Given that the predominant approach to clinical trial design is within a frequentist paradigm and that analysing only Bayesian researchers would not have answered our intended research question, the term ‘Bayesian’ was not used in the participant information sheet. As such, it is possible that we missed people who had a potentially strong view on such methods. However, we felt this was preferable in order to sample a wider range of participants. Given that none of our participants had experience in using Bayesian methods to incorporate prior information, an interesting further study might involve identifying and interviewing trialists who do have such experience. This study might explore how and why these trialists have used Bayesian approaches and how any barriers might have been overcome.

## Conclusions

In conclusion, trialists recognise that more formal use of external evidence could be advantageous over current approaches in some areas, particularly to inform parameters other than the treatment effect, for which very limited information may be available from the trial, and useful as sensitivity analyses. Trialists do however note that there are still many barriers to such use in practice. Clear guidance, user-friendly software and accessibility to a repository of data might increase uptake of Bayesian approaches in practice.

## Supplementary Information

**Additional file 1: Table S1.** COREQ checklist. **Figure S1.** Example of topic guide.

## Data Availability

N/A

## Abbreviation

RCT: Randomised controlled trial; NIHR: National Institute for Heath Research; PIS: participant information sheet
