# Supplementary material for: Use of external evidence for design and Bayesian analysis of clinical trials: a qualitative study of trialists’ views
Source: Trials. 2021 Nov 8;22:789. doi: 10.1186/s13063-021-05759-8 (PMC8577005; doi:10.1186/s13063-021-05759-8)
Supplement: Supplementary file 1 — Additional file 1: Table S1. COREQ checklist. Figure S1. Example of topic guide. [file 13063_2021_5759_MOESM1_ESM.docx]

**Supplementary material**

Contents

Table S1 COREQ checklist

Figure S1 Example of topic guide

**Table S1 COREQ checklist**

|  | **No** | **Item** | **Guide questions/description** | **Comment** |
| --- | --- | --- | --- | --- |
| **Domain 1: Research team and reflexivity** | | | | |
| **Personal characteristics** | 1 | Interviewer/facilitator | Which author/s conducted the interview? | GC |
|  | 2 | Credentials | What were the researcher’s credentials? e*.g. PhD, MD* | MSc |
|  | 3 | Occupation | What was their occupation at the time of the study? | PhD Student in Trials Methodology |
|  | 4 | Gender | Was the researcher male or female? | Female |
|  | 5 | Experience and training | What experience or training did the researcher have? | GC has 2 years’ experience working as a trial statistician at a clinical trials unit. GC has also been qualitative workshops and short courses. GC is also working with an experienced qualitative researcher DE. |
| **Relationship with participants** | 6 | Relationship established | Was a relationship established prior to study commencement? | Yes. Since GC has worked in clinical trials for 2 years prior to starting the PhD she was able to invite several people to interview who met the inclusion criteria. |
|  | 7 | Participant knowledge of the interviewer | What did the participants know about the researcher? *e.g. personal goals, reasons for doing the research* | GC introduced herself, explained the purpose of the research and provided an information leaflet about the study. GC did not go into detail about her PhD until after the interview so that her views were not pushed onto the participants. |
|  | 8 | Interviewer characteristics | What characteristics were reported about the interviewer/facilitator? *e.g. Bias, assumptions, reasons and interests in the research topic* | GC stated that all the answers given could be positive or negative regarding the use of previous evidence in trials. |
| **Domain 2: study design** | | | | |
| **Theoretical framework** | 9 | Methodological orientation  and theory | What methodological orientation was stated to underpin the study? *e.g. grounded theory, discourse analysis, ethnography, phenomenology, content analysis* | Data were analysed thematically using techniques of constant comparison derived from grounded theory methodology. |
| **Participant selection** | 10 | Sampling | How were participants selected? *e.g. purposive, convenience, consecutive, snowball* | Purposeful and snowball. |
|  | 11 | Method of approach | How were participants approached? e*.g. face-to-face, telephone, mail, email* | GC contacted researchers by email |
|  | 12 | Sample size | How many participants were in the study? | 13 statisticians and 3 clinical academics |
|  | 13 | Non-participation | How many people refused to participate or dropped out? Reasons? | Of the people asked to participate, one person who was asked and said they would take part, then could not find time. |
| **Setting** | 14 | Setting of data collection | Where was the data collected? *e.g. home, clinic, workplace* | Interviews were held at the participants workplace. |
|  | 15 | Presence of non-participants | Was anyone else present besides the participants and researchers? | No |
|  | 16 | Description of sample | What are the important characteristics of the sample? *e.g. demographic data, date* | Participants’ full details are provided in Table 1, and key information is provided in the methods section |
| **Data collection** | 17 | Interview guide | Were questions, prompts, guides provided by the authors? Was it pilot tested? | Topic guides were developed (based on the study aims and relevant literature along with DE) to ensure that discussions covered the same basic issues but with sufficient flexibility to allow new issues of importance to the informants to emerge. A pilot interview was conducted, however as all questions were deemed relevant, this was used as participant data. As analysis progressed, the topic guide adapted to enable exploration of emerging themes and a log of amendments was recorded. A separate topic guide was used for clinicians which included similar question but rephrased. |
|  | 18 | Repeat interviews | Were repeat interviews carried out? *If yes, how many?* | No repeat interviews were carried out |
|  | 19 | Audio/visual recording | Did the research use audio or visual recording to collect the data? | Interviews were audio-recorded. |
|  | 20 | Field notes | Were field notes made during and/or after the interview? | GC kept a few notes about each interview detailing the overall tone and the key points. |
|  | 21 | Duration | What was the duration of the interviews? | Interviews lasted an average of 54 minutes (range = 37 - 79 minutes). |
|  | 22 | Data saturation | Was data saturation discussed? | Data collection continued until GC and DE were confident that saturation had been reached. |
|  | 23 | Transcripts returned | Were transcripts returned to participants for comment and/or correction? | Transcripts were not returned to participants for comments or corrections |
| **Domain 3: analysis and findings** | | | | |
| **Data analysis** | 24 | Number of data coders | How many data coders coded the data? | GC initially coded the data, and emerging themes were discussed with DE, with reference to the raw data and coding frame*.* Double coding between GC and HJ was used for three transcriptions in total, with reference to the raw data. In that time, we were happy that concerns in interpretation of coding frame was reached. |
|  | 25 | Description of the coding tree | Did authors provide a description of the coding tree? | A description of the coding tree is not provided in the article |
|  | 26 | Derivation of themes | Were themes identified in advance or derived from the data? | Themes were derived from the data |
|  | 27 | Software | What software, if applicable, was used to manage the data? | NVivo (version 11) was used to analyse the data |
|  | 28 | Participant checking | Did participants provide feedback on the findings? | Results were not sent out for response validation |
| **Reporting** | 29 | Quotations presented | Were participant quotations presented to illustrate the themes / findings? *Was each quotation identified? e.g. participant number* | The interpretation of each theme is supported by illustrative quotes. Each quote is identified by a participant code |
|  | 30 | Data and findings consistent | Was there consistency between the data presented and the findings? | There is consistency between the data presented and the findings (see Table 2) |
|  | 31 | Clarity of major themes | Were major themes clearly presented in the findings? | The themes are clearly presented in the findings |
|  | 32 | Clarity of minor themes | Is there a description of diverse cases or discussion of minor themes? | Description of diverse cases and where minor themes occurred between participant groups are discussed |

**Figure S1 Example of topic guide**

Opening

- Thanks/ PIS (broadly), Aim (purpose), voluntary, stop at any time, can’t answer any questions just move on, completely confidential/ Any questions - Consent form - Start recording!

| **Job role** | | **Group** | **Years in profession** | | **Types of trials** | **Type of unit** | **Affiliation** |
| --- | --- | --- | --- | --- | --- | --- | --- |
|  | |  |  | |  |  |  |
|  | **Question** | | | **Probes** | | | |
| **1** | **Background/ role** | | | How *long* have you worked there? *anywhere else*? | | | |
| **2** | **Previous evidence in a trial?** | | | Before a trial has started – concepts/ Design/ your role | | | |
| **3** | **Methods using to analyse trials** | | | How do you *choose* which methods to use? *vary* by the type of trial?  Are they using *relatively simple* analyses or more *complex analyses*? | | | |
| **4** | **Previous evidence in analysis stage** | | | Examples of where previous evidence has been used in the analysis stage of a trial? / Do these examples vary by type of trial, type of outcome / Where do you think this previous evidence should come from? | | | |
| **5** | **Bayesian methods to analyse a clinical trial** | | | Initial thoughts/ any experience? Any colleagues? Prior distributions  informative prior distributions/ non-informative / subjective priors  Advantages/disadvantages | | | |
| **6** | **Informative prior distributions to inform parameters (such as the treatment effect) in the analysis stage?**  *See information sheet for further explanation if required* | | | ANY experience of using informative priors/ colleagues  Advantages/disadvantages/resource waste?  Power a new trial based on the impact it can have on a meta-analysis?  What do you think about using an objective prior (based on a synthesis of previous evidence)? | | | |
| **7** | **Bias adjustment** | | | At the end of a trial are you always confident you can believe the results or do you think they may be at a risk of bias or many biases? E.g. if you couldn’t blind the patients.  Example if knew average amount of bias  Any examples where it may not be possible to do some of these things that the trial was less likely to get funded? | | | |
| **8** | **Parameters during the analysis which are poorly estimated or need more power?** | | | What types of parameters are usually poorly estimated?  Do you know *why* they are poorly estimated? What do you do when parameters are poorly estimated? How do you report parameters which are poorly estimated? Did you find a solution to improve estimation? Do you think previous evidence could have helped? Would you use previous evidence if it helped estimation? Adverse events? | | | |
| **9** | **Would you use informative priors on parameters in the analysis of a trial?** | | | Do you know of any situations where using informative priors could be useful? Or where it may not be applicable? How are new methods implemented? permission ? sensitivity analysis? | | | |

Closing

Checks understanding of any outstanding points/ Thank them for their time/ Answer further questions, Ask who else we can speak to – contacts?

Date: XX Version number: v1.XX
